# The Role of the Person Focused IARA Model in Reducing Anxiety and Improving Body Awareness and Illness Management in Diabetics with Acquired Lipodystrophy: A Mixed-Method Study

**DOI:** 10.3390/jpm12111865

**Published:** 2022-11-08

**Authors:** Francesca Viglino, Maha Sellami, Fabio Broglio, Paola Scuntero, Anna Maria Padovan, Claudia Maulini, Guglielmo Beccuti, Nicola Bragazzi, Massimiliano Barattucci, Goran Kuvačić, Andrea De Giorgio

**Affiliations:** 1 Department of Medical Sciences, University of Turin, 10123 Torino, Italy; 2 Physical Education Department, College of Education, Qatar University, Doha P.O. Box 2713, Qatar; 3 Kiara Association Onlus, 10123 Torino, Italy; 4 Department of Movement Sciences and Wellbeing, University of Naples Parthenope, 80133 Napoli, Italy; 5 Laboratory for Industrial and Applied Mathematics (LIAM), Department of Mathematics and Statistics, York University, Toronto, ON M3J 1P3, Canada; 6 Department of Human and Social Sciences, University of Bergamo, 24129 Bergamo, Italy; 7 Faculty of Kinesiology, University of Split, HR-21000 Split, Croatia; 8 Faculty of Psychology, eCampus University, 22060 Novedrate, Italy

**Keywords:** anxiety, psychological indices, emotions, patient-centered, person-centered, compliance

## Abstract

Background: Lipodystrophy is one of the most frequent complications in people with diabetes following subcutaneous insulin therapy, and poor management can lead to several problems, such as impaired glycemic control and adherence to therapy, anxiety, and depression. Poor injection technique represents the main risk factor for lipodystrophies. In order to enhance the patient’s insulin injection technique to heal lipodystrophy, improve psychological indices, and promote involvement in their health and care, the efficacy of emerging person-centered care called the IARA model was tested. Methods: A total of 49 patients were randomly allocated to the IARA group (Experimental; *n* = 25) or standard education (Control; *n* = 24). The following questionnaires were used in a mixed-method design: (i) State Anxiety Scale; (ii) Beck Depression Inventory; (iii) Italian Summary of Diabetes Self-Care Activities. An ad hoc open-ended questionnaire was structured for the qualitative analysis. Finally, photos were taken in order to verify if injection sites were changed until the follow-up at 12 months. The number of patients who participated until the completion of the study was 17 in the IARA and 11 in the Control group. Results: State anxiety was significantly reduced in people who followed IARA to follow-up at 3 and 6 months (*p* < 0.05). The IARA group also demonstrated better compliance in blood glucose monitoring and foot-care compared to Control at follow-up at 12 months. The management of insulin injections dramatically improved in participants who received IARA intervention. Conclusions: IARA could be considered an effective strategy to improve well-being and compliance in people affected with diabetes mellitus and lipodystrophy complications.

## 1. Introduction

Diabetes mellitus (DM) is one of the most common chronic diseases in the world, and it is characterized by high levels of glucose in the blood due to a lack of insulin secretion and/or action. According to the World Health Organization [1], 422 million people worldwide have diabetes. In Italy, based on the latest available statistics [2], the prevalence of diagnosed diabetes is approximately 5.9%, with more than 3.5 million people affected.

It is well known that achieving and maintaining optimal glycemic control and glycated hemoglobin (HbA1c) levels is necessary to reduce the risk of acute and chronic complications, such as macrovascular and microvascular diseases and avoid premature death [3,4].

In all types of DM, treatment must include different types of intervention, such as pharmacological treatment and an adequate lifestyle, as well as an education program for DM self-management. Insulin therapy has been indispensable in type 1 diabetes (T1DM) since the diagnosis, but it is also indicated in the treatment of type 2 diabetes (T2DM) at different stages of the disease [5]. In addition, a correct injection technique is of primary importance to ensure optimal insulin action [6]. It is estimated that more than 60% of people with diabetes develop lipodystrophies related to it [7].

Lipodystrophy is one of the most frequent complications in people with DM following subcutaneous insulin therapy, and poor management can lead to several problems, the most important of which is impaired glycemic control among patients [8,9]. Patients’ poor injection technique represents the main risk factor for lipodystrophies, which are characterized by glycemic oscillations that lead to hyper- or hypoglycemia [6].

The role of education in patients with lipodystrophy is quite well known [10,11,12,13] because it can improve injection techniques for patients. However, this syndrome remains a challenge to face to improve both the patients’ quality of life and insulin therapy management. Literature has widely demonstrated that adherence to care is essential to patients in order to ameliorate the path of treatment of the illness [14] and that a scarce adherence to care in DM can lead to poor management of illness. Poor perception of self-efficacy in curing one’s disease leads to psychological problems such as anxiety [15], depression [16], and distress [17]. To improve adherence to care, it is important to implement careful therapeutic education, which is particularly undeniable in treating DM [18].

The so-called IARA model is emerging in the literature as a useful educational tool for improving both adherences to care and psychological indices. [19,20,21]. IARA is person-focused care [22] that—briefly, since widely demonstrated and described elsewhere—consists of a model that uses both some specific tools (awareness drawing, qualities, imagery, counseling, “3A rules”) and tools especially designed for the pathology to be addressed. For example, through IARA, in chronic obstructive pulmonary disease [21], a respiratory system model (bronchus and lungs) was built using a bottle, straws, and balloons to raise awareness of how we breathe in by person affected by COPD. IARA was also very helpful in headache [23], gastroesophageal reflux [24], in the care pathway of the treatment of hip [19] and knee prostheses [20]. For all these reasons, although IARA focuses on the whole person, its peculiarity is that it has tools useful to bring out needs and solutions from the person himself. Moreover, IARA was proved helpful in ameliorating the quality of life of healthcare professionals [25], which is important [26] because their well-being is able to improve patients’ mental health [27].

As mentioned, poor management of injection techniques leads to a general worsening of DM. Therefore, to improve the injection technique by patients and subsequently their quality of life and to heal lipodystrophy, the authors chose to verify the efficacy of IARA in outpatient clinic and compared it with usual educational care in people affected by DM.

## 2. Materials and Methods

### 2.1. Participants

**Figure 1:**
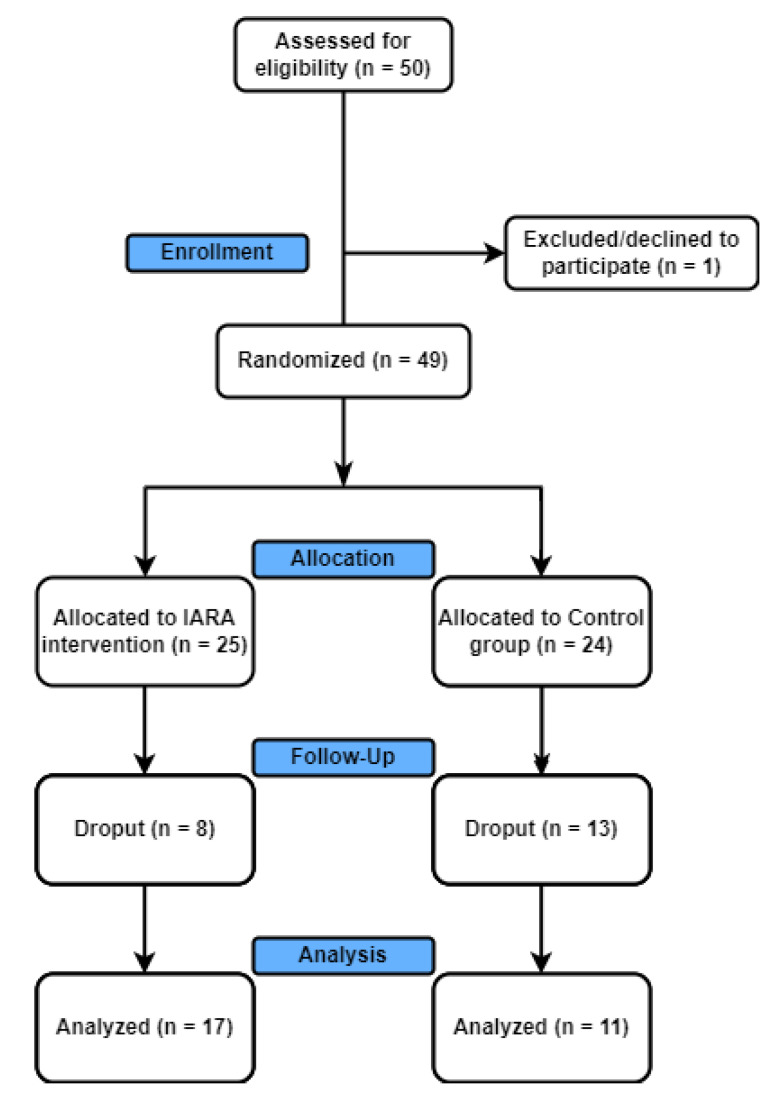
Consort diagram. The final sample, excluding dropouts, was composed of 17 patients for IARA and 11 for Controls.

Fifty participants with T1DM and T2DM were recruited from outpatient diabetes clinics at the San Giovanni Antica Sede Hospital, Turin, Italy, between October 2019 and January 2020. The hospital is a nationally accredited diabetes center. It comprises health professionals specializing in diabetes care, including endocrinologists, nurse practitioners, diabetes nurse educators, and a range of allied health professionals (dieticians, psychologists, podiatrists). Participants were randomly assigned to Control (Control; 66.55 ± 12.65 years) and Experimental group (IARA; 54.88 ± 22.72 years). The final sample, excluding dropouts, was composed of 17 (M = 8; F = 9) patients for IARA and 11 (M = 8; F = 3) for Control group (Figure 1).

### 2.2. Data Collection Procedures

#### 2.2.1. Standard Procedure (Control)

Once lipodystrophy had been identified, the physician sent the patient allocated to this group to follow the usual therapeutic education meetings. As already described in literature [28] these meetings aim to improve the insulin therapy and site rotation technique. During the first meeting of 60 min, the questionnaires were administered, and filled out.

#### 2.2.2. IARA Intervention

**Figure 2:**
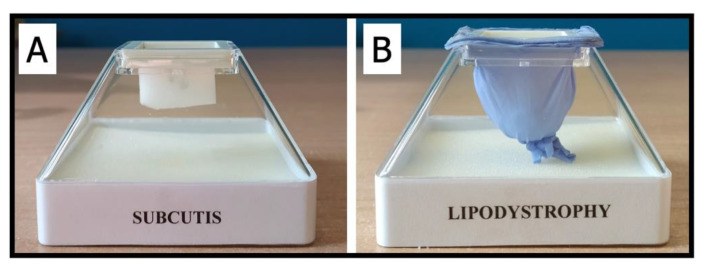
Representation of the pyramid was used to raise participants’ awareness of what happens at the injection site. (**A**) The use of a piece of gauze simulated the subcutis. (**B**) With the use of a piece of glove and cotton, lipodystrophy was simulated.

As aforementioned in the Introduction section, alongside specific IARA tools two more ad hoc have been added for diabetic people affected by lipodystrophy. In particular, were created: (i) a pyramid (Figure 2) that simulates healthy subcutaneous tissue and a tissue affected with lipodystrophy; (ii) photos of one’s abdomen that was expected that will allow the person to become aware of the entire abdomen surface available for injection sites.

*First meeting*. In the first meeting (T0), approximately 30 min long, the questionnaires were first administered and filled out. Afterward, without providing any type of information regarding lipodystrophy, a diabetes nurse educator (DNE) asked the person to draw the injection points used for insulin therapy on his body with a demographic pencil for a week.

*Second meeting*. During this one-hour meeting, one week after T0, the DNE aimed to raise awareness of the participants using some tools of transpersonal psychosynthesis theory [29] and the 3A rule (in Italian: *Accoglienza*, *Ascolto*, *Accettazione incondizionata positiva*; that is: hospitality, listening, positive unconditional acceptance [29]).

At the beginning of the meeting, a photo of the injection sites that the person had drawn was taken. The photo was then transferred to a computer, and observing the image with the DNE, the person gained a new perception of the adequacy of the rotation of the injection sites.

The DNE, listening openly with a positive attitude, let the person’s verbalization finish and then introduced the conscious drawing to help him achieve a degree of awareness that could identify any mistakes.

The “*awareness drawing*”—specificity of IARA—consisted of three phases: (i) try to draw lipodystrophy based on the knowledge possessed and/or that acquired during the meeting; (ii) observe the drawing and try to describe the sign of lipodystrophy; (iii) find and design a solution aimed at improving lipodystrophy.

With the patient’s new awareness, the DNE observed the photo with him again and asked him to make further observations about the adequacy or otherwise of the injection sites. At this point, the ad hoc tool (Figure 2) was used to make the participant aware of the effects of the injection (which had to be done inside the subcutis to avoid impaired insulin absorption) and of the safety of using the needle (overcoming fears about damaging tissues and internal structures).

The meeting ended with a request to re-perform, with the new knowledge acquired, the mapping of the injection points, with an educational reinforcement on recognizing symptoms and treating hypoglycemia.

*Third meeting*. The meeting took place 21 days after T0 and using the same reception principles already described, the DNE immediately gave the person the opportunity to narrate his experience of the disease and of any behavioral change regarding the injection technique. With this new awareness, a second photo of the injection sites was taken to show the rotation made by the participant in the previous week. Afterward, the downloaded glycemic values were evaluated with the patient, and then both agreed on changes in the new lifestyle. The participant was then asked to think about his positive qualities and list them or stimulate them using a dedicated tool [21,30]. The meeting ended with a request to continue to rotate the injection sites appropriately and to expand the list of his attributes.

*Fourth meeting*. The meeting took place 30 days after T0 and began by providing the participant with the opportunity to narrate his experience, the achievements reached during the month, and any obstacles and changes applied (e.g., variation of insulin units). In addition, he was asked to share the positive qualities that might have been added to the list written during the previous meeting. Subsequently, the glycemic diary was re-analyzed by being compared with events, lifestyles, and changes that occurred in the rotation of the sites; the DNE paid particular attention to the autonomy achieved by the participant, reinforcing the obtained goals. All aforementioned meetings conducted by DNE, were supervised by a clinical psychologist. Psychologist did not take part to the meetings, but conducted briefing and debriefing sessions in order to support DNE.

### 2.3. Measures

#### 2.3.1. Quantitative Measures

To study the effect of IARA training compared with Control training, a qualitative and quantitative study was performed and divided as follows: T0 = recruitment, submission of questionnaire; T1 = 3 months follow-up; T2 = 6 months follow-up; T3 = 12 months follow-up. The following questionnaires were used:

*State Anxiety Scale (STAI-Y1)* [31]: a 20-item self-report questionnaire on a 4-point Likert scale (from 1 = not at all to 4 = very much so; original Cronbach’s alpha = 0.954) to assess participant’s current state of anxiety.

*Beck Depression Inventory (BDI)* [32]: the 13-item version was used, which asks participants to rate the extent to which they are experiencing each of the 13 common symptoms of depression. The questionnaire is based on a 4-point Likert scale (starting from 0, with 3 representing the highest severity), with 39 as the maximum score. In agreement with the literature, a cut-off score of 9/10 seems best suited for indicating the presence/absence of depression where the questionnaire is used as screening [33].

*Italian Summary of Diabetes Self-Care Activities (SDCA)* [34]: a brief self-report questionnaire of diabetes self-management characterized by a core set of 11 items regarding the diabetes regimen: Nutrition (2 items), Diet (2 items), Exercise (2 items), Blood Glucose Monitoring (2 items), Foot-Care (2 items), and Smoking Status (1 item). The strengths of the 11 core items of the revised SDCA include their brevity and ease of scoring. Scores are calculated for each of the five regimen areas assessed by the SDCA. Regarding items 1–10, a 0–7 Likert scale was used to highlight the number of days per week in which a patient follows (or not) the regimen. Regarding the Smoking Status, a single item was used: 0 = nonsmoker, 1 = smoker; when smoker, the patient had to provide the number of cigarettes smoked per day.

#### 2.3.2. Qualitative Measures

An ad hoc open-ended questionnaire [35] was structured to highlight the differences between IARA and Control groups. Questions were the following: “Can you describe your experience of a person who has lipodystrophy?”; “Can you describe your experience with respect to the care of the nurses and doctors at this center?”; “Can you describe what you found positive and negative about your experience”. The participants were asked to fill out the questionnaire after 3 and 12 months. From the content analysis, two categories are body image perception and disease awareness improvement.

The content analysis was conducted using the paragraph as a textual unit. Based on both the literature and the content analysis [21,30,36,37] the system of categories was chosen as follows:

- Macro category: Categories/Indicators;
- IARA Categories: Body image perception (BP)/Disease awareness (DA);
- IARA Indicators: Perception, Cognition (BP)/Engagement/Management/Autonomy (DA).

From the content analysis, two indicators emerged for the *Body image perception* category: Perception (i.e., the way sensory information is organized, interpreted, and consciously experienced) and Cognition (i.e., mental process with people acquiring knowledge and understanding through thoughts, experience, and the senses) [38]. Furthermore, three indicators emerged for *Disease awareness*: Engagement (includes clinicians’ efforts to engage patients in the therapeutic process through education and counseling and patients’ engaging themselves through disease awareness, adoption of lifestyle changes, and medication adherence). Management/Autonomy. These indicators include: (i) clinicians’ efforts to engage patients in the therapeutic process through education and counseling and patients’ engaging themselves through disease awareness, adoption of lifestyle changes, and adherence to treatment; (ii) involving patients in the decision-making process in their care process; (iii) the need for independence, volition, and a sense of purpose in life [39,40,41].

#### 2.3.3. Photos

Photos are used in order to improve the awareness of the injection site. This choice was made only for people belonging to the experimental group because it is not a procedure foreseen in normal therapeutic education. The participants were asked to draw a circle around all injection sites during the experimental period and follow-up to improve their awareness of injection sites. The first photo was taken one week after T0. Then, three more photos were taken: the second, third, and fourth ones at T1, T2, and T3, respectively. The photos taken were then used to check if people had improved rotation of the injection sites.

### 2.4. Statistical Analysis

Mean differences between the IARA and Control group in baseline data were compared using an unpaired Student’s *t*-test. The normality of the analyzed data was checked using the Kolmogorov–Smirnov test. After confirmation of assumptions for parametric tests, data were presented as mean and standard deviations (M ± SD). A two-way mixed-design between-within 2 × 4 ANOVA was conducted in order to detect differences between IARA and Control group (factor Group) in each of the applied variables before (T0), 3 months (T1), 6 months (T2) 12 months (T3) after the intervention (factor Time) and their interactions. A one-way repeated measures ANOVA analyzed the simple main effect among the 4-time points in each group. Assumption of sphericity was checked with Levene test, and if violated, Greenhouse-Geiser correction of degrees of freedom was applied. Analysis of covariance (ANCOVA) was applied to those variables in which Time or Group had interaction effects to assess the group differences at the 6- and 12-months. For the multiple comparisons, post-hoc analysis with Bonferroni correction was used if significant main effects were detected. Effect sizes are presented as partial eta-squared (η2p) to determine the meaningfulness of the results. As suggested by Cohen [42], threshold values to effect size were 0.01 (small), 0.06 (medium), and 0.14 (large). The level of significance was fixed to *p* < 0.05. Statistical analyses were carried out using SPSS 24 statistical package.

## 3. Results

Unpaired Student’s *t*-test showed no significant group differences (Control vs. IARA) for any measured variables or characteristics at the baseline of the research.

A two-way mixed-design ANOVA (Table 1) revealed (a) a group effect for anxiety levels; (b) a time and a group effect for diet self-care activities; (c) a group and a time x group effects for blood glucose monitoring activities; (d) an interaction time x group for foot-care activities. Age and sex were not controlled as covariates because there were no differences in these data at the pre-test. Moreover, the covariates were not linearly related to the dependent variable at each level of the independent variable.

A one-way repeated measures ANOVA (Table 2) analyzed the simple main effects among the time points in IARA and Control. These results revealed a slight significant time effect in the IARA group: anxiety levels remained unchanged through time in Control and decreased in IARA. Moreover, a significant time effect on diet for both groups was found (highest in IARA): post-hoc analysis (Bonferroni) revealed a higher level of self-care activities concerning diet in T0 and T1 compared to T2 in IARA participants. Finally, the analysis showed that both blood glucose monitoring and foot-care activities decreased in Controls whereas remaining stable in IARA. However, post-hoc analysis did not reveal any significant differences between time points.

**Figure 3:**
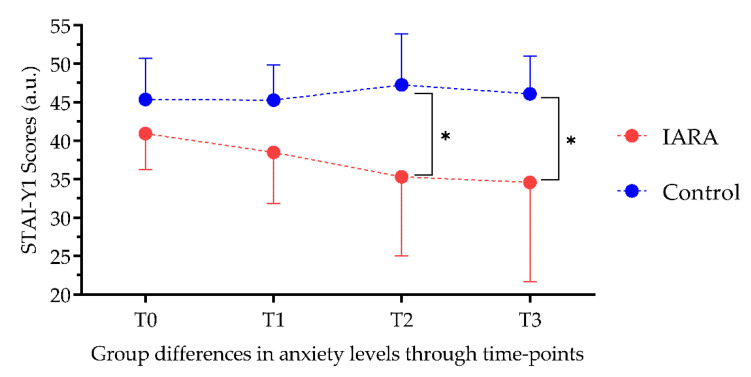
State anxiety was significantly reduced in people who followed the IARA model. STAI-YI—State Anxiety Scale; T0—baseline; T1—3-month follow-up; T2—6-month follow-up; T3—12-month follow-up; *—*p* < 0.05.

**Figure 4:**
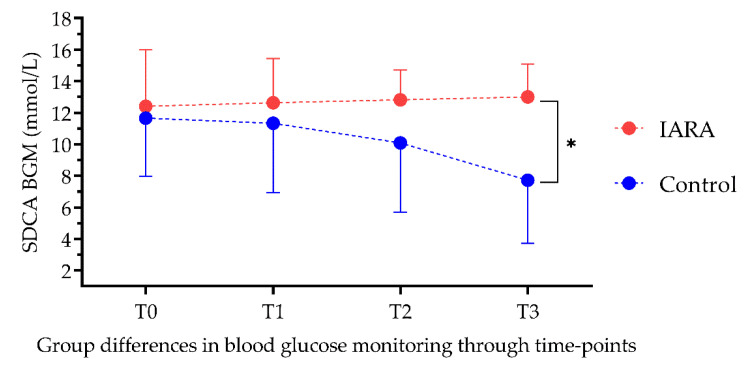
People who followed the IARA model demonstrated better compliance in blood glucose monitoring compared to Control. SDCA BGM—Italian Summary of Diabetes Self-Care Activities/blood glucose monitoring; T0—baseline; T1—3-month follow-up; T2—6-month follow-up; T3—12-month follow-up; *—*p* < 0.05.

**Figure 5:**
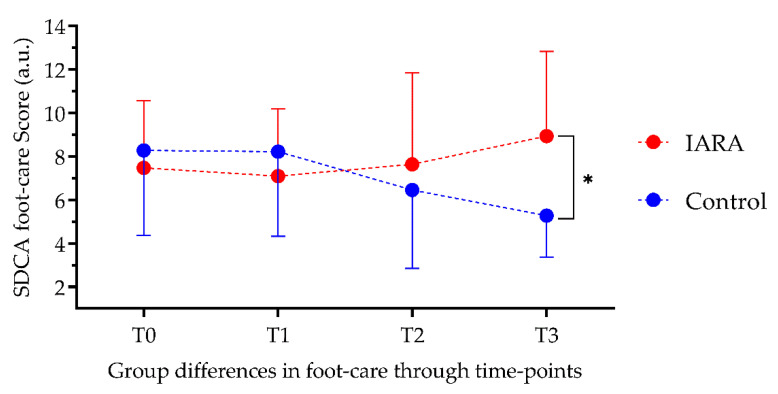
People who followed the IARA Model demonstrated better foot-care compliance than Control. SDCA foot-care—Italian Summary of Diabetes Self-Care Activities/foot-care; T0—baseline; T1—3-month follow-up; T2—6-month follow-up; T3—12-month follow-up; *—*p* < 0.05.

ANCOVA showed that groups were significantly different after 6 and (F = 11.79; *p* < 0.001) 12 months in STAI-Y1 (F = 8.8; *p* < 0.01; Figure 3), Blood Glucose Monitoring (F = 14.1; *p* < 0.001; Figure 4) and Foot-Care levels (F = 8.59; *p* < 0.01; Figure 5) after 12 months. No significant differences were found in SDCA diet (T2: F = 1.99 and T3: F = 0.131, *p* = >0.05)

**Figure 6:**
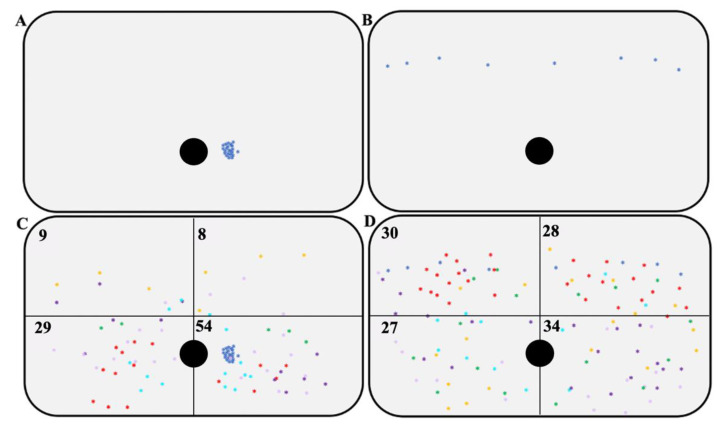
The distribution of self-injection sites. (**A**) Distribution of self-injections by a case belonging to the IARA one week after T0; (**B**) Distribution of self-injections by the same case in A at 12 months follow-up (T3); (**C**,**D**) Distribution of self-injections of IARA one week after T0 (**C**), and at 12 months follow-up (T3; (**D**)). Numbers at the corner represent the self-injections in that part of the abdomen, and it is possible to observe the most homogeneous distributions of self-injections at T3 rather than T0. Numbers at the corner represent the self-injections in that part of the abdomen, and it is possible to observe the most homogeneous distributions of self-injections at T3 rather than T0.

As aforementioned, in the first meeting, the participants were asked to draw the injection points used for insulin therapy on their body with a demographic pencil for a week. The photos taken as described in the Section 2.3.3 were used to compare if the injection points changed during the experimental period and follow-up. From each participant’s photos, the injection sites’ distribution was derived (Figure 6A,B is an example). Additionally, the abdomen was arbitrarily subdivided into four parts (Figure 6C,D)—paying particular attention to the different anatomical proportions of the participants—overlapping all injection sites and finding a more homogeneous distribution of the injections at T3. As shown in Figure 6, it is possible to highlight a more homogeneous and extended distribution of the injections at the 12 months follow-up, demonstrating that IARA dramatically improved in the management of insulin injections.

Regarding the category of *body image perception*, the qualitative analysis revealed that it is possible to highlight how participants affirmed to have better understood how injection should be done concerning their own body, acquiring more awareness about it. This was evidenced by these explicative sentences:

- Thanks to this model, [the insulin effects] are much clearer; I know what happens to my body when the injections are injected badly;
- By knowing my body and the right injection insulin sites everything becomes freer. My body and my mind are “aligned” with my blood sugar;
- Since starting the IARA model, I have realized that I was unaware of some parts of my body that I did not consider;
- The model I followed with the nurses was clearer to me when they explained my body’s reaction to the daily insulin intake and the strategic points to perform it precisely.

From the second qualitative analysis, i.e., *disease awareness*, participants showed an increased awareness of the sentinel symptoms of diabetes, and the importance of both physical exercise and treatment of the disease, as declared by the participants:

- Initially, I did not notice the hypoglycemia, and therefore I was always a little anxious; but now that I am more aware and realize it, I am much calmer;
- This model enticed me to go to the gym and/or walk, hoping my blood sugar would be maintained well;
- The model taught me the opportunity to vary the area where I inject insulin to prevent the thickening of the subcutaneous tissue;
- The positive side of my experience is that it has helped me grow and become responsible.

## 4. Discussion

This study demonstrated that IARA could be considered a useful tool for improving patients’ psychological indices and promoting their involvement in diabetes management and care. Literature has already demonstrated the effectiveness of prevention and educational strategies on DM management [43,44]. Among DM complications, lipodystrophy is one of the most frequent [8,9], with a prevalence ranging from 28.7% [45] to 64.4% [7]. Due to the reduced vascularization and capillary density of the localized lipodystrophy, insulin is absorbed in a variable and unpredictable way.

Blanco et al. [7] studied 430 patients affected by diabetes treated with insulin for at least one year, demonstrating rates of frequent unexplained hypoglycemia and glucose variability were more than six to seven times higher in patients with lipodystrophy than in those without. There was also a significant correlation between the presence of lipodystrophy, the reuse of the needle, and a strong association with the site’s rotation: 98% of the patients with lipodystrophy did not rotate or rotate incorrectly. A significant increment of mean insulin daily dose was also found. Similar results were found by Vardar and Kizilci [46], who analyzed data from 215 DM patients who had been using insulin for at least two years. The study showed that the amount of time insulin had been used, the frequency of changing injection sites, and the frequency of changing needles can dramatically influence the development of lipodystrophy.

A Worldwide Injection Technique Questionnaire Study including 13,289 participants with DM found that people with lipodystrophy had higher HbA1C values, on average by 0.55%, a higher number of hypoglycemic events, greater glycemic variability, greater risk of ketoacidosis, and a greater total amount of insulin dose administered, on average 10 IU [28]. Furthermore, psychological aspects are crucial in treating chronic diseases [24,47,48,49], and DM is no exception [17,50]. Literature describes several educational approaches to manage DM [51]. These approaches encompass patients’ self-management [52], awareness [53], empowerment [54], and compliance [18]. Most of the DM education is performed by nurses, physicians, or dietitians, and interventions can include counseling sessions, brief lessons on the pathophysiology, diet, exercise, medication compliance, and glucose monitoring in DM. It has been demonstrated that education influences the HbA1C level better when performed face-to-face rather than in a group. In particular, counseling done by a diabetes educator (or a team of them) delivered in a variety of formats may reduce HbA1C levels by 0.2% to 0.8% compared with usual care alone.

The effect of individual diabetes education compared to group education or usual care on HbA1C levels was investigated in a Cochrane review of nine randomized controlled trials, including 1359 adults with T2DM with an 18-month follow-up [55]. In patients with an average baseline HbA1C level greater than 8%, individual diabetes education resulted in a greater HbA1C decrease than usual care. However, the presence of lipodystrophy highlights that, at least in part, the failure of education leads to scarce management of blood sugar with all the consequences it generates. For these reasons, an effective educational approach that avoids lipodystrophies or reverses them is decisive in treating DM. Authors have successfully tested IARA, characterized by several tools taught to nurses and physicians to use during planned patient meetings [25]. The present study has proved that IARA can effectively manage DM, ameliorate psychological indices, foot-care compliance, and monitor blood glucose. Because disease awareness is closely linked with patient engagement, an essential factor for adherence to the therapeutic regimen [56], it is possible to infer that IARA has improved this aspect.

Moreover—and this is no less important—people belonging to the IARA group have dramatically changed the injection sites, which is considered the most important thing to learn in order to avoid lipodystrophies. IARA is an approach that responds to basic principles of patient-centered care [57], based on a different relationship between healthcare professionals and patients and, therefore, is a quality of personal, professional, and organizational relationships. However, the philosophy behind IARA allowed, for example, to develop ad hoc educational tools, such as the “pyramid” (Figure 2), and effectively overcome the use of other types of tools, such as the injection rotation guide [58]. Actually, the manufacturer’s rigorous guide is not suitable for all patients, even just because the abdomen’s surface can change dramatically from person to person. For this reason, IARA overcomes patient-centered care by shifting toward person-focused care [59].

As confirmed by the qualitative analysis, the use of the pyramid and the photos allowed the participants to achieve a significant leap in their body awareness. It is important to highlight that body perception can influence health-related behaviors—such as physical activity, nutrition, and other behaviors, which are important components in DM management [60,61]. IARA participants better perceived their body image and a general understanding of their health and specific risk knowledge [54]. It has already been demonstrated that body image and lipodystrophy influence each other [62], and for this reason qualitative interviews were chosen only for the IARA group. Indeed, changes were expected only in the group in which body awareness was promoted. Body awareness and body image perceptions are particularly important in people with weight problems [53,63], and it changes based on ethnicity [64] and gender [65,66] in DM. Based on the results, IARA could be considered an innovative model in T2DM education and care, and it will be necessary to increase the sample to verify its effectiveness on biological indices such as, for example, HbA1C.

### Limitations of the Study

Further quantitative and qualitative studies are necessary to show the full effectiveness of the IARA model in diabetes management because, due to the small sample, data are not generalizable. However, patients’ results on the management of diabetes and the impact of IARA on psychological indices are highly encouraging.

## Figures and Tables

**Table 1:** Results of the two-way mixed-design ANOVA—within/between participant’s effects and their interactions.

Variable	Time		Group		Time × Group	
	F	η2	F	η2	F	η2
STAI-Y1	1.99	0.07	15.37 **	0.37	1.98	0.06
BDI	1.21	0.05	0.13	0.01	1.34	0.04
SDCA Nutrition	1.54	0.06	0.92	0.03	1.99	0.05
SDCA Diet	14.77 **	0.36	4.44 *	0.2	0.34	0.02
SDCA Exercise	1.15	0.04	2.16	0.17	1.21	0.04
SDCA Blood glucose monitoring	1.28	0.05	4.36 *	0.21	2.98 *	0.12
SDCA Foot-Care	0.66	0.02	0.256	0.01	4.89 *	0.21

Legend: Data are presented as mean and standard deviation (SD); STAI-YI—State Anxiety Scale; BDI—Beck Depression Inventory; SDCA—Italian Summary of Diabetes Self-Care Activities; F—f test; η2—effect size (eta squared) *—*p* < 0.05; **—*p* < 0.001.

**Table 2:** Results of one-way repeated measures ANOVA with Bonferroni post-hoc test.

Variable	Group	T0	T1	T2	T3	F	η^2^	Post-Hoc
		Mean (SD)	Mean (SD)	Mean (SD)	Mean (SD)			
STAI-Y1	CONT	45.36 (5.35)	45.27 (4.6)	47.27 (6.6)	46.09 (4.9)	0.33	0.06	n.s.
	IARA *	40.94 (4.7)	38.47 (6.6)	35.29 (6.8)	34.58 (8.2)	3.72 *	0.32	1 > 4
BDI	CONT	5.22 (6.8)	4.66 (5.4)	4.44 (3.8)	4.68 (6.51)	0.22	0.1	n.s.
	IARA	5.23 (5.1)	6.1 (4.3)	5.0 (4.2)	5.7 (6.7)	2.55	0.35	n.s.
SDCA Nutrition	CONT	11.66 (4.4)	9.55 (4.8)	7.66 (4.3)	10.77 (4.4)	8.36 *	0.8	1 > 3
	IARA *	11.0 (3.7)	10.52 (3.7)	8.88 (4.1)	11.23 (2.9)	3.53 *	0.43	n.s.
SDCA Diet	CONT	10.45 (2.8)	9.36 (2.7)	6.0 (2.7)	8.7 (2.9)	3.93 *	0.28	1 > 3
	IARA *	8.76 (2.9)	7.7 (3.0)	5.8 (3.4)	6.44 (2.4)	11.31 **	0.41	1, 2 > 3
SDCA Exercise	CONT	4.11 (3.5)	5.33 (4.3)	5.77 (3.9)	4.88 (4.2)	1.38	0.34	n.s.
	IARA *	3.7 (3.5)	4.47 (3.9)	4.46 (3.3)	3.8 (3.7)	0.35	0.05	n.s.
SDCA Blood glucose monitoring	CONT	11.66 (3.7)	11.33 (4.4)	10.09 (4.2)	7.72 (4.9)	3.28 *	0.24	n.s.
	IARA *	12.41 (3.6)	12.64 (2.8)	12.82 (4.6)	13.0 (2.2)	0.53	0.03	n.s.
SDCA Foot-Care	CONT	8.27 (3.9)	8.22 (3.9)	6.49 (3.2)	5.27 (3.7)	3.34 *	0.25	n.s.
	IARA *	7.47 (3.1)	7.09 (3.1)	7.64 (3.2)	8.94 (3.2)	1.81	0.11	n.s.

Legend: Data are presented as mean and standard deviation (SD); STAI YI—State Anxiety Scale; BDI—Beck Depression Inventory; SDCA—Italian Summary of Diabetes Self-Care Activities; F—f test; η2—effect size (eta squared); post-hoc—Bonferroni correction; n.s.—not significant; *—*p* < 0.05; **—*p* < 0.001.

## Data Availability

The datasets generated and analyzed for the current study are available from the corresponding author, A.D.G., upon reasonable request.
